# Pharmacokinetic Study in Mice of Galphimine-A, an Anxiolytic Compound from *Galphimia glauca*

**DOI:** 10.3390/molecules19033120

**Published:** 2014-03-12

**Authors:** Rodolfo Abarca Vargas, Alejandro Zamilpa, Francisco Alarcón Aguilar, Maribel Herrera-Ruiz, Jaime Tortoriello, Enrique Jiménez-Ferrer

**Affiliations:** 1 Centro de Investigación Biomédica del Sur, Instituto Mexicano del Seguro Social (IMSS), Argentina No. 1, Col. Centro, Xochitepec 62790, Morelos, Mexico; E-Mails: rodolfo.abarca@uaem.mx (R.A.V.); azamilpa_2000@yahoo.com.mx (A.Z.); cibis_herj@yahoo.com.mx (M.H.-R.); jtortora2@yahoo.es (J.T.); 2 Doctorado en Ciencias Biológicas y de la Salud, División de Ciencias Biológicas y de la Salud, Universidad Autónoma Metropolitana-Iztapalapa, San Rafael Actlixco No. 186, Col. Vicentina, Iztapalapa 09340, Mexico; E-Mail: aaaf@xanum.uam.mx

**Keywords:** *Galphimia glauca*, pharmacokinetics, phytopharmaceutical, galphimine-A, anxiety

## Abstract

The aim of this study was to obtain pharmacokinetic data for the anxiolytic compound galphimine-A (G–A) from *Galphimia glauca*. G–A is the most abundant anxiolytic compound in this plant, while Galphimine-E (G–E) is the most abundant galphimine, but inactive. G–E was transformed chemically into G–A. The pharmacokinetic study was carried out in ICR mice, which were orally administered a single 200 mg/kg dose of G–A. Samples of blood and brain were taken at different times after administration of G–A. Previously, we established the validation of methods for determining the concentration of G–A. The G–A was detected in plasma 5 min after oral administration, and its concentration reached 2.47 μg/mL. Data from concentration-time curves allowed us to establish the main pharmacokinetic parameters in two models: one- and/or two-compartment. C_max_ values were 3.33 and 3.42 μg/mL respectively, likewise AUC_0→1440 min_ were 1,951.58 and 1,824.95 μg/mL·min. The G–A in brain tissue was noted to cross the blood-brain barrier, reaching C_max_ 2.74 μg/mL, T_max_ 81.6 min, and then drop gradually to 0.32 μg/mL detected at 24 h. The presence of G–A in brain tissue, confirmed that this anxiolytic compound can access the target organ and acts directly on the CNS.

## 1. Introduction

**Figure 1:**
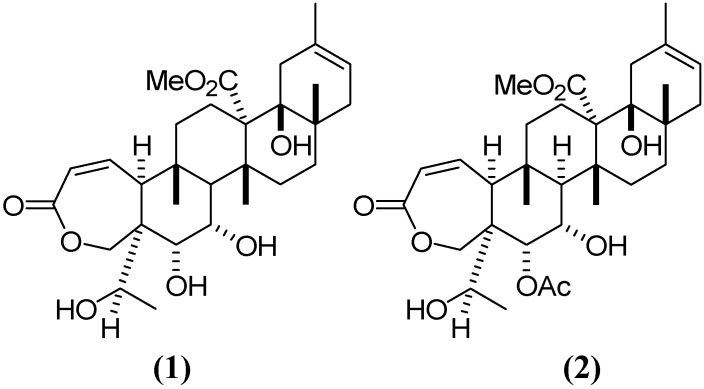
Chemical structure of galphimines: (**1**) G–A, (**2**) G–E.

*Galphimia glauca* Cav. (Malpighiaceae), commonly known in Mexico as “calderona amarilla”, has long been used in Mexican traditional medicine for the treatment of mental disorders [1]. Extracts obtained from the aerial parts of this plant have pharmacological effects on the central nervous system (CNS) [2]. In particular, anxiolytic activity has been shown in behavioral animal models for the extract obtained from *G. glauca* [3]. Some of the active compounds, generically known as galphimines, were obtained by bio-guided chemical separation. Of these, the nor-secotriterpenes galphimine-A (G–A) and galphimine-B (G–B) showed the highest anxiolytic activity [4]. In a double-blind clinical trial, a phytopharmaceutical elaborated with a standardized *Galphimia glauca* extract proved to be effective and tolerable in patients with generalized anxiety disease [5]. The active extract obtained from *G. glauca* contains a third compound known as galphimine-E (G–E). Although G–E is not active, it is present at a higher concentrations in *G. glauca* [6]. All the galphimines have similar chemical structures, the only difference between G–A and G–E being the presence of an acetyl moiety at the C7 position (Figure 1). Thus, it is possible to transform G–E into G–A in order to obtain larger amounts of the active compound for testing. In plant extracts, the concentration of G–B, the most potent anxiolytic galphimine, is very low, and the isolation of this compound in its pure form is methodologically complicated. Therefore, the present pharmacokinetic study was exclusively focused on G–A.

Substances with pharmacological effects on the CNS, as in the case of active compounds from *G. glauca*, must reach the brain to cause their effect, so G–A has to cross the blood-brain barrier in order to exert its effects. Therefore, the objective of this work was to determine the transference processes of G–A at the blood-brain interphase. Pharmacokinetic data of G–A were determined in mice orally administered this active compound, which was obtained either naturally by plant extraction, or chemically by transformation of naturally isolated G–E into G–A.

## 2. Results and Discussion

### 2.1. Chromatographic Analysis of G–A and G–E

**Figure 2:**
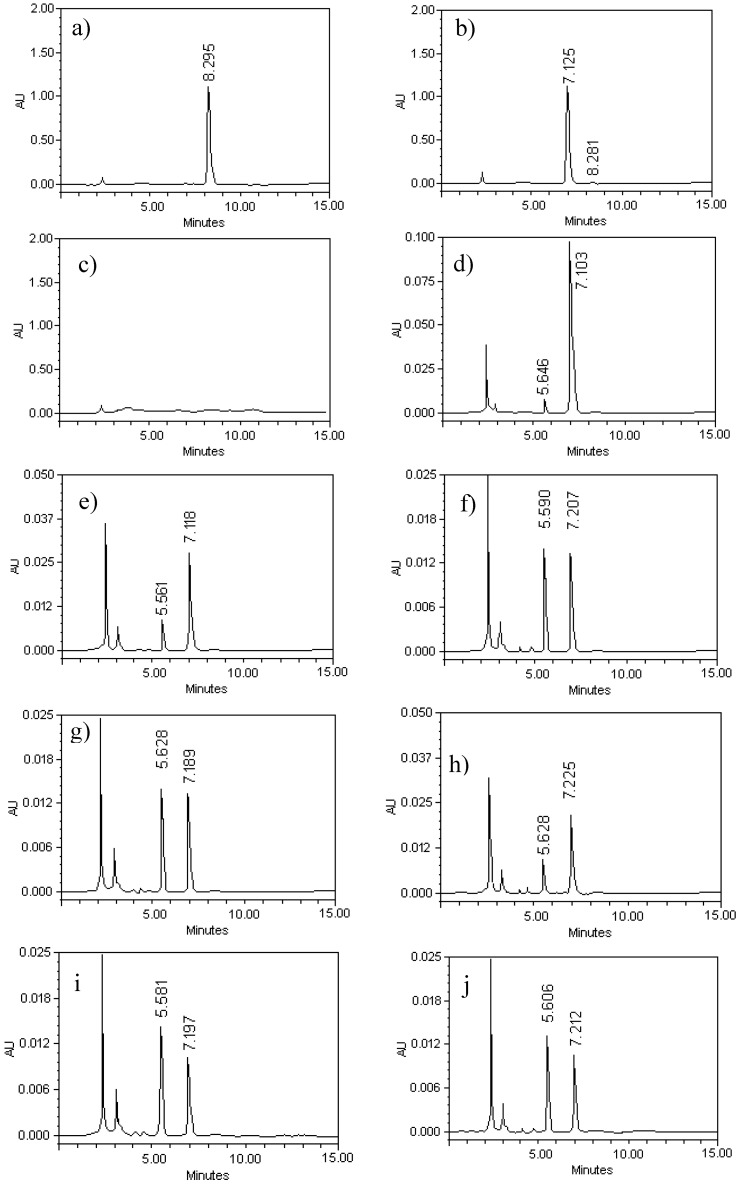
HPLC chromatograms for galphimine-A (G–A), which represent the process of production, purification and validation of process of quantitation of both plasma and brain. The analyses were performed in a Waters 2695 HPLC. A Cromotith Performace^®^ RP-18e column (100 mm × 4.6 mm) was used and operated a 25 °C. The mobile phase consisted of acetonitrile:water (35:65, 1 mL/min). Panel (**a**) and (**b**), show the monitoring of the transformation of G–E (RT = 8.29 min) to G–A (RT = 7.12 min). The chromatogram (**c**) shows the untreated mouse plasma. The chromatograms displayed in panels (**d**), (**e**), (**f**) and (**g**), show the internal standard peak and galphimine-A in decreasing concentration in plasma (62.5, 15.62, 3.9 y 0.97 µg/mL). The retention times were about 5.5 and 7.2 respectively. The chromatograms displayed panels (**h**), (**i**) and (**j**), showing the internal standard peak and galphimine-A in brain homogenate (12.5, 3.12 and 0.78 µg/mL). The retention times were again about 5.5 and 7.2 respectively.

Figure 2, panels (a) and (b) illustrate the chromatographic profile of G–A and G–E. Retention times (RT) for G–A and G–E were 7.12 and 8.29 min, respectively. The presence of the acetate group at the C7 position of G–E increased the retention time; this fact was useful in order to monitor the transformation of G–E to G–A. Due to this difference in RT, G–A of high purity was later obtained. The yield of the reaction reached values above 85%.

### 2.2. Method Validation

#### Standardization of the Chromatographic Process

The methods were validated using the following criteria: for linearity and sensitivity, the data were found to be linear over a concentration range of 0.24 to 62.50 µg/mL in plasma samples and a range 0.78 to 12.5 µg/mL in brain samples. The regression equation for plasma was (y) = 15,285(x) + 2,065, with the correlation coefficient *r* = 0.9995; in the brain tissue homogenate the standard curve was described by the equation (y) = 20,285(x) + 917, *r* = 0.9993. If r > 0.999, this indicates good linearity. The limited of quantitation (LOQ) was 0.12 µg/mL for plasma and 0.09 µg/mL for brain tissue, with a value of precision (RSD) < 20%.

Under these chromatographic conditions, the number the theorical plates was 8,100 and the resolution of G–A was 125 peaks/min. Any degree of interference by endogenous plasma or brain tissues constituents with G–A and internal standard was ruled out by analyzing the chromatograms of both the blank plasma and brain tissue.

Regarding the specificity of the procedure, the internal standard and G–A were eluted around 5.5 and 7.2 min, respectively (Figure 2d). A good separation of internal standard and G–A was obtained under the specified chromatographic conditions. There is no disturbance from the background signals either in the plasma or the brain after the extraction with methanol for protein samples that have been exhaustively dehydrated by lyophilization (Figure 2c).

The intra- and inter-day assay accuracies, recoveries and stability of G–A were estimated at three concentrations (0.80, 4.00 and 10.00 µg/mL) in both plasma and brain tissue (Table 1). The intra and inter-assay accuracies were expressed as the percent differences between the measured concentration and nominal concentration. Intra-assay precision and accuracy were calculated using (*n* = 6) determination for each concentration of the spiked plasma sample during a single analytical run. Inter-assay precision and accuracy were calculated using replicate (*n* = 6) determinations of each concentration made on three separate days. Accuracy (%Bias) = [(C_obs_ − C_nom_) / C_nom_] × 100. The precision (relative standard deviation; RSD) was calculated from the observed concentrations as follows: RSD = [standard deviation (SD) / C_obs_] × 100. Accuracy (Bias) and precision (RSD) values ± 15% covering the range of actual experimental concentration were considered acceptable [7].

**Table 1:** Quantification of G–A in mouse plasma and brain tissue, determination of precision and accuracy (N = 6).

Matrix Sample	Nominal Concentration (μg/mL)	Observed Concentration (μg/mL) ± S.D.	Accuracy (% Bias)	RSD (%)
Plasma Intra-day	0.80	0.77 ± 0.04	−3	5.2
	4.00	3.96 ± 0.09	−4	2.3
	10.00	9.98 ± 0.97	−2	9.7
Plasma Inter-day	0.80	0.75 ± 0.07	−5	9.3
	4.00	3.94 ± 0.10	−6	2.5
	10.00	10.01 ± 1.04	1	10.4
Brain tissue Intra-day	0.80	0.78 ± 0.06	−2	7.7
	4.00	3.94 ± 0.12	−6	3.0
	10.00	10.01 ± 0.73	1	7.3
Brain tissue Inter-day	0.80	0.77 ± 0.08	−3	10.4
	4.00	3.96 ± 0.11	−4	2.8
	10.00	10.02 ± 0.17	2	1.7

The intra-day and inter-day precision (% RSD) in mouse plasma and brain tissue were <15%. The recovery was approximately 85% to 98% in the plasma and 94% to 97% in brain tissue (Table 2).

**Table 2:** Recovery yield of G–A from mouse plasma and tissue brain (*n* = 6).

Matrix Sample	Spiked Concentration (μg/mL)	Recovery Index ± S.D.	RSD (%)
Plasma	0.80	0.854 ± 0.11	13.2
	4.00	0.874 ± 0.09	10.3
	10.00	0.978 ± 0.14	14.3
Tissue brain	0.80	0.951 ± 0.09	8.5
	4.00	0.968 ± 0.08	8.3
	10.00	0.943 ± 0.13	13.8

The extraction efficiency of G–A was determined by analyzing sets (*n* = 6) of 0.80, 4.00 and 10.00 μg/mL samples for both plasma and brain tissue, representing low, medium and high QCs, respectively. Recovery was calculated by comparing the peak areas of G–A added into blank samples and extracted using the lyophilization to insolubilize to proteins with those obtained G–A spiked directly into post-protein insolubilization by lyophilization at three QC concentration levels.

The stability of G–A is shown in Table 3. The precision for freeze/thaw samples ranged from 3.5% to 10.1% for plasma and 5.0% to 9.6% for brain tissue, and the accuracy ranged from −1.00% to 0.25% for plasma and 3.6% to 9.6% for brain tissue, respectively. The results indicated that G–A was stable in either plasma or brain tissue for three cycles when stored at −70 °C and thawed to room temperature. The precision for the autosampler stability ranged from 6.0% to 10.9% for plasma and 4.3% to 12.6% for brain tissue, and the accuracy ranged from −1.75% to 10.00% for plasma and 91.24% to 96.67% for brain tissue, respectively. These results suggested that G–A could be analyzed for over 8 h in the autosampler tray at 4 °C with acceptable levels of precision and accuracy. The precision and accuracy for long–term stability samples ranged from 7.2% to 8.8% and −5.7% to 6.50% for plasma and 9.4% to 13.9% and −10.0% to 4.30% for brain tissue, respectively. The results of long-term storage stability data indicated that the plasma and brain tissue samples were stable at −70 °C for over 1 month. The results of stability experiments showed that no stability-related problems occurred during sample storage, extraction or chromatographic processes for G–A in plasma and brain tissue samples.

**Table 3:** Stability profile of G–A under different handling conditions.

Matrix Sample	Handling Conditions	Nominal Concentration μg/mL)	Observed Concentration (μg/mL) ± S.D.	AccuracyBias (%)	RSD (%)
Plasma	3 Freeze/thaw cycles (−70 °C)	0.80	0.79 ± 0.08	−1.00	10.1
		4.00	4.01 ± 0.14	0.25	3.5
		10.0	9.93 ± 0.67	−0.70	6.7
	Autosampler stability (4 °C; 8 h)	0.80	0.83 ± 0.05	3.75	6.0
		4.00	3.93 ± 0.43	−1.75	10.9
		10.0	10.10 ± 0.85	10.00	8.4
	Long–term stability (−70 °C; 1 month)	0.80	0.77 ± 0.06	−3.00	7.8
		4.00	4.26 ± 0.31	6.50	7.2
		10.0	9.43 ± 0.83	−5.70	8.8
Brain tissue	3 Freeze/thaw cycles (−70 °C)	0.80	0.83 ± 0.08	3.75	9.6
		4.00	4.36 ± 0.34	9.00	7.8
		10.0	10.36 ± 0.52	3.60	5.0
	Autosampler stability (4 °C; 8 h)	0.80	0.87 ± 0.11	8.75	12.6
		4.00	3.60 ± 0.33	−10.0	9.1
		10.0	9.83 ± 0.43	−1.70	4.3
	Long–term stability (−70 °C; 1 month)	0.80	0.72 ± 0.10	−10.00	13.9
		4.00	3.73 ± 0.41	−6.75	10.9
		10.0	10.43 ± 0.98	4.30	9.4

The stability of G–A in mouse plasma and brain tissue homogenate was performed by analyzing samples (*n* = 6) of QC in concentrations of 0.80, 4.00 and 10.00 μg/mL. The determination of stability of the triterpene considered the conditions which would subject the samples, these include: stability during the process of freeze/thaw, residence time in the autosampler, and long–term storage. The concentrations obtained from all stability studies were compared with the freshly prepared QC samples, and the percentage concentration deviation was calculated. The analytes were considered stable in mouse plasma and brain tissue homogenate when the concentration difference between the freshly prepared samples and the stability testing samples was less than 15%.

### 2.3. G–A Plasma Levels and Brain

The data obtained from the determination of G–A concentration in plasma and brain tissue by HPLC (Table 4), were used in the PKSolver program [8]. After a single oral administration of 200 mg/kg of G–A, the behavior of the concentration-time data allowed to determine the main pharmacokinetic parameters under the two models, one- and two-compartment (Table 5). C_max_ values were 3.33 and 3.42 μg/mL respectively, likewise AUC_0→1440min_ were 1951.58 y 1824.95 μg/mL·min. G–A was fast absorbed (Table 3), its presence was detectable in plasma 5 min after oral administration and reached a concentration of 2.47 μg/mL. The fall in G–A plasma concentration continued for 24 h (corresponding to the monitoring time). Regarding the performance of G–A in the brain compartment, it reached C_max_ of 2.74 μg/mL, T_max_ 81.6 min (Table 6), and then dropped gradually to 0.32 μg/mL detected at 24 h. Such a good distribution of G–A in the brain is certainly a result of the lipophilic properties of the molecule, which allows it to easily cross the blood-brain barrier.

**Table 4:** G–A concentration variations in ICR mouse plasma and brain in the first 24 h after oral administration of a single dose of 200 mg/kg of G–A.

	G–A Concentration (μg/mL)	
Time (min)	Plasma	Brain	
5	2.47 ± 0.43	0.00	
10	2.57 ± 0.52	0.88 ± 0.33 *	
15	2.79 ± 0.79	1.48 ± 0.58 *	
30	3.70 ± 0.70	3.44 ± 0.50	
60	3.44 ± 0.35	3.01 ± 0.59	
120	2.95 ± 0.43	2.50 ± 0.58	
240	2.33 ± 0.42	2.18 ± 0.48	
480	1.44 ± 0.37	1.78 ± 0.37	
720	0.96 ± 0.21	0.55 ± 0.18 *	
1440	0.31 ± 0.02	0.32 ± 0.14	

Data presented as means ± S.E.M. with *n* = 8. ANOVA and post hoc Tukey test. * *p* <0.05 compared to plasma data.

**Figure 3:**
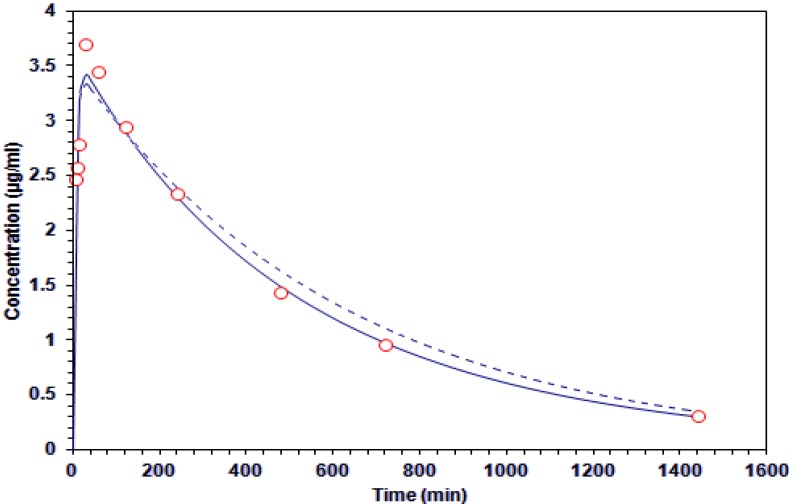
Concentration-time curve of galphimine A in plasma. The experimental data (○) and estimates of the application of one-compartment (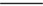) and two-compartment (-----) models.

The data were fit both to one compartment and two compartment models (Figure 3). The equations describing the compartments are as follows:

C*_p_* = C_1_**e**^−*kd*t^ + C_2_**e**^−*ka*t^ (one compartment model)

C*_p_* = C_1_**e**^−αt^ + C_2_**e**^−βt^ + C_3_**e**^−*kd*t^ (two compartment mode)

where C*_p_* is the plasma concentration (μg/mL); *k_d_*, β, disposition rate constant (min^−1^); *k_a_*, absorption rate constant (min^−1^); C_1_, C_2_ and C_3_, coefficients (µg/mL). The goodness of fit were assessed with Sum of Squares (SS), Akaike Information Criteria (AIC), Schwarz Criteria (SC) and Determination coefficient (R^2^) (Table 4), to establish the pharmacokinetics of GA after oral dosage of 200 mg/kg. Data from the plasma concentration *versus* time were analyzed under the two proposed model (one and two-compartment). It was established that the one-compartment model was fitted best according to the value of AIC and SC (Table 5). However, the value of R^2^ and the behavior of the data in Figure 3, suggest that the two-compartment model can be considered.

The Table 5 shows *k*_10_ values of G–A was 0.0016 and 0.0017 min^−1^ one- and two-compartment, respectively.

**Table 5:** Pharmacokinetic parameters of G–A in plasma ICR mice, *n* = 8.

Parameter	One-Compartament Value	Two-Compartament Value	Units
A	3.50	1.83	μg/mL
B	---	1.82	μg/mL
k_a_	0.182	0.164	1/min
k_10_	0.0016	0.0017	1/min
k_12_	---	0.00017	1/min
k_21_	---	0.00195	1/min
t_1/2ka_	3.79	4.22	1/min
α	---	0.0025	1/min
β	---	0.0014	1/min
V/F	57.59	55.49	(mg/kg)/(μg/mL)
CL/F	0.092	0.098	(mg/kg)/(μg/mL)/min
T_max_	26.16	27.35	min
C_max_	3.33	3.42	μg/mL
AUC_0→∞_	2170.15	2030.23	μg/mL·min
AUC_0-1440 min_	1951.58	1824.95	μg/mL·min
MRT	630.40	620.45	min
Diagnostics			
SS	0.7217	0.6331	
R^2^	0.9885	0.9899	
AIC	−6.1575	−4.6565	
SC	−5.2498	−3.1436	

**Table 6:** Pharmacokinetic parameters of G–A in brain tissue ICR mice, *n* = 8.

Parameter	Value	Unit
A	2.98	μg/mL
k_a_	0.051	1/min
k_10_	0.0008	1/min
t_1/2 ka_	13.50	1/min
V/F	68.16	(mg/kg)/(μg/mL)
CL/F	0.057	(mg/kg)/(μg/mL)/min
T_max_	81.59	min
C_max_	2.74	μg/mL
AUC_0→__∞_	3519.23	μg/mL·min
AUC_0-1440 min_	1912.49	μg/mL·min
MRT	1218.86	min

The concentration-time data after a single oral administration of 200 mg/kg of G–A conformed to one- and two-compartment models. A and B: G–A plasma concentration in central and peripheral compartment, respectively; k_a_: apparent first-order absorption rate constant; k_10_: apparent first-order elimination rate constant from central compartment; k_12_: apparent first-order transfer rate constant from central compartment to peripheral compartment; k_21_: apparent first-order transfer rate constant from peripheral compartment to central compartment; t_1/2ka_: absorption half time; α and β: empirical constants corresponding to the coefficients of the exponents of the values of A and B; V/F: apparent volume of distribution; CL/F:apparent clearance; T_max_: time to maximal concentration; C_max_: maximal concentration; AUC_0→∞_ y AUC_0-1440 min_: Area under the plasma concentration curve from 0 to infinity and form 0 to 1440 min, respectively; MRT: mean residence time; SS: sum of square; R^2^: correlation index; AIC: Akaike information criteria; SC: Schwarz criterion.

The plasma area under the G–A concentration time curve throughout the measurement period reached values of 2,170 and 2,030 μg·min·mL^−1^.

The anxiolytic effect of galphimines already had been reported [3], indicating that G–A and G–B are the most active pharmacologically [4]. G–E is much more abundant that the other galphimines, although inactive [6]. In this report we show that deacetylation of G–E converts it into GA, with a conversion rate higher than 80%. This simple reaction may represent an important improvement in the production process of an anxiolytic phyto-medicine, standardized concentration of active galphimines. G–A had anxiolytic effects with a 15 mg/kg dose administered intraperitoneally [4], and in the present work the dose chosen was 200 mg/kg, due to the fact the administration pathway was oral and to make sure it would be detected when monitored in the plasma and brain samples. Regarding the pharmacokinetic behavior of G–A, and with the intention of measuring the concentration of this triterpene in the brain this organ was lyophilized and the water removed was taken into account to infer the amount of G–A in this system and report it in concentration units (µg·mL^−1^). 

The data analysis shows that G–A its absorbed quickly, as it was detected in plasma within 5 min of its oral administration, with a slow decay of plasma concentration, since it was still detected at 24 h after that the test started. This suggests slow plasma clearance, similar to other triterpenes like glycyrrhetinic acid, which is the active compound in licorice [9].

The anxiolytic activity of galphimines implies that they can access the brain compartment, however in this work evidence that galphimines cross the blood-brain barrier is provided. This is based on the fact the G–A concentration in the brain compartment reached 2.74 µg/mL within 81.6 min, and gradually decays within 24 h. These parameters indicate a good distribution of G–A in the brain that is certainly a result of the lipophilic properties of the molecule, which allow it to easily cross the blood-brain barrier.

There are no antecedents concerning the pharmacokinetic behavior of the galphimines, not even of similar compounds. There are reports about another triterpene, glycyrrhetinic acid. Considering the mass exchange between the compartments, it was observed that the value of *k*_10_ (elimination rate constant) obtained for G–A, in a model of one-compartment was of 0.0016 min^−1^, and for the two-compartment model it was 0.0017 min^−1^, while for the triterpene glycyrrhetinic acid in mice (administered with 100 mg/kg, orally) and the one-compartment model the corresponding value of *k*_10_ was of 0.0006 min^−1^ [10], which is evidently different. In contrast, the *k*_10_ value for G–A in mice and the *k*_10_ value for glycyrrhetinic acid (0.0012 min^−1^) in humans, show the same order of magnitude [11]. 

The area under the curve for G–A was comparable in the two models analyzed compared to the AUC_0→∞_ found for oral administration of glycyrrhetinic acid in mice: 2,454 µg·min·mL^−1^ [10]. Finally, the G–A concentration decay rate was determined as a function of time in the brain compartment. According to AIC values, the model that best fits these pharmacokinetic parameters is the one-compartment one.

## 3. Experimental

### 3.1. Chemicals

HPLC grade acetonitrile, methanol, and water (Merck, KGaA, Darmstadt Germany), trifluoroacetic acid (Mallinckrodt Inc., Phillispburg, NJ, USA), analytical potassium bicarbonate, orthophthalaldehyde, *n*-hexane, ethyl-acetate, and methanol (Merck, Naucalpan de Juárez, México state, Mexico), as well as β-ciclodextrin (Sigma-Aldrich, Saint Louis, MO, USA) were used.

### 3.2. Plants Material

Aerial parts of *Galphimia glauca* Cav. (Malpighiaceae) were collected from a two-year-old controlled crop located at Xochitepec, Morelos, Mexico (18°47'40.70'' N y 99°11'49.27'' W, 1,185 m.a.s.l.). Plant material was authenticated by Abigail Aguilar Contreras, head of the IMSSM Herbarium (Mexican Social Security Institute Herbarium), and one specimen was deposited at the Herbarium for future reference.

### 3.3. Extraction and G–A Purification

The material was dried under dark conditions at room temperature. Thus, 25 kg of dried material were milled with an electrical device (Pulvex, Mexico city, Mexico) until <4 mm particles were obtained, which were then extracted with 9 volumes of *n*-hexane for 24 h at room temperature (extract was discarded). Next, the material was extracted three times with 9 volumes of methanol for 24 h at room temperature. The methanolic extract was concentrated in a rotary evaporator under reduced pressure and subjected to liquid-liquid separation using water and ethyl acetate. The organic phase fraction was separated and the solvent evaporated. This fraction was then separated in an activated carbon column and products were eluted with 1:1 *n*-hexane-ethyl acetate. After the collected products were added to a normal phase gravity-flow column (2.5 × 70 cm) containing silica gel (Merck, 70:230 mesh), and *n*-hexane-ethyl acetate-methanol 49:49:2 was used as mobile phase; 250 mL aliquots were obtained, which were concentrated at reduced pressure. Finally, pure compounds were obtained by preparative layer chromatography.

To transform the obtained G–E into G–A, the dried product containing G–E was dissolved in a saturated KHCO_3_ solution (2 mg/mL of G–E). The mixture was kept at room temperature with shaking (100 rpm) for 24 h. The product was then added to a silica gel gravity flow column (Merck 70:230 mesh, 0.9 × 20 cm). This column was first eluted with ethyl acetate (12 mL approx.) in order to remove the non-transformed G–E. Then the column was eluted with methanol (36 mL approx.). The product was analyzed by HPLC to verify the purity of the transformed compound. The purity of the obtained G–A was greater than 97%. Identity of the galphimines was confirmed by comparison of ^1^H-, and ^13^C-NMR spectroscopic data with previously published values [6]. 

### 3.4. Animals

ICR albino mice of 30–36 g were used (Harlan México, D.F.). Animals were housed in groups of 8 per cage, maintained under laboratory conditions at 25 °C, with light/dark schedule 12 h (lights on at 07:00 a.m.) and free access to water and food (pellets from Harlan Rodent Lab Diet). Mice were allowed three weeks to adapt to the laboratory environment before experiments. All studies were performed in accordance with the Mexican Official Regulation NOM-062-ZOO-1999 (Technical Specifications for Production, Care and Use of Laboratory Animals). The project was approved by the local health research committee (IMSS), the institution which authorizes the ethical use of animals (registered under the number R-2006-1701-20). The least number of animals and time of observation required to obtain consistent data were used.

### 3.5. Preparation of Standard and Internal Standard Stock Solution

The G–A standard stock solution was prepared by dissolving 50 mg of G–A in 50 mL methanol (HPLC grade) to obtain a nominal concentration of 1 mg/mL. The internal standard (IS) stock solution was prepared by dissolving 0.20 mg of *O*-phthalaldehyde (OPA) in a 100 mL of methanol (HPLC grade) to obtain a nominal concentration of 2 μg/mL. All the stock solutions were maintained at 4 °C until use.

### 3.6. HPLC Calibration Curve of G–A

Samples of plasma with G–A standard (0.24, 0.98, 3.90, 15.62, 62.50 μg/mL) were prepared by addition to the plasma with appropriate amounts of the stock solution prepared above. Quality control (QC) samples were used to determine accuracy and precision of the method, and were independently prepared at low (0.80 μg/mL), medium (4.00 μg/mL) and high (10.00 μg/mL) concentrations in the same manner. The I.S. was added to each standard sample before sample processing. All plasma samples were lyophilized before to quantification of G–A.

Samples of brain homogenate with G–A standard (0.78, 1.56, 3.12, 6.25, 12.50, μg/mL) were prepared by adding the appropriate amount of standard stock solution prepared above. QC samples were used to determine accuracy and precision of the method, and were independently prepared at low (0.80 μg/mL), medium (4.00 μg/mL) and high (10.00 μg/mL) concentrations in the same manner. The IS was added to each standard sample before sample processing. All brain tissue samples were lyophilized before to quantification of G–A.

### 3.7. Experimental Design of G–A Administration and Blood and Brain Sample Collection

Eighty-eight mice were randomly divided into 11 groups with eight individuals each. All animals were orally administered with 200 mg/kg of G–A dissolved in 5% β-cyclodextrin solution. Under ether-induced anesthesia, blood samples were obtained from the retroorbital sinus with heparinized cannula at different times (0, 5, 10, 15, 30, 60, 120, 240, 480, 720, and 1,440 min) after G–A administration. The volume of the blood samples was of 700 to 1000 microliters, which were collected in heparinized centrifuge microtubes, after the blood was centrifuged for 5 min at 1,720 × *g*.

Plasma samples were frozen at −70 °C, and lyophilized after. The obtained dry product was used to extract and measure the amount of G–A. After obtaining blood samples, all animals were perfused with isotonic saline solution (ISS) via cardiac puncture; the femoral vein was used to eliminate the perfusion liquid, until it appeared clear. Subsequently, the brain was removed. The brains were weighed fresh and frozen at −70 °C and then lyophilized. Once dry, the brains were weighed again to determine the volume of water contained in each brain. Dry tissue was ground in a mortar, and G–A was later extracted.

### 3.8. Quantification of G–A in Plasma and Brain Samples

Dry pulverized samples (plasma and brain) were extracted with methanol (HPLC grade, Merck). The extracts were filtered with a 0.45 μm, 13 mm Teflon membrane (Millex^®^-LCR), and 20 μL of filtrate were injected into the HPLC equipment. Measurements were performed in a Waters 2695 HPLC, with diode array detector (Waters 2996) and the Empower Pro 1.0 software. A Chromolith Performance^®^ RP-18e 100 × 4.6 mm (Merck KGaA Darmstadt, Germany) was used. The mobile phase consisted of a mixture of acetonitrile:water (55:45, 1 mL/min). G–A concentration was obtained by comparison with a pre-built calibration curve at λ = 220 nm.

### 3.9. HPLC Calibration Curve of G–A

In order to determine G–A concentrations contained in different samples, a calibration curve was built with the HPLC system. An initial solution of 1 mg/mL G–A in methanol (HPLC grade) was used to prepare a series of solutions by successive dilutions to establish decreasing concentration of G–A (62.5, 15.6, 3.9, 0.98, 0.24 μg/mL), and injected into the HPLC by triplicate.

### 3.10. Method Validation

Methods validations were performed according to guidelines by Food and Drug Administration (FDA) for analytical methods validation [7].

### 3.11. Linearity and Sensitivity

For the evaluation of the linearity of standard calibration curve, the determinations of G–A in plasma and brain samples were performed on three independent days using fresh preparations. The calibration curves were prepared over a linear range of 0.24 to 62.5 μg/mL for plasma and 0.78 to 12.5 μg/mL for brain samples. Each calibration curve was compared against a double blank sample with and without internal standard and five calibrator concentrations.

Each calibration curve was constructed by plotting the analyte to internal standard peak area ratio (y) against analyte concentrations (x). The calibration curves were fitted using a least square linear regression model (y) = m(x) + b using Microsoft Office Excel 2010 software. The resulting m, b parameters were used to determine back-calculated concentrations, which were the statistically evaluated. All calibration curves of G–A were constructed before the experiments with correlation values of at least 0.9995.

### 3.12. Specificity

The specificity was defined in reference to two conditions, first non-interference term when G–A was not retained by the endogenous components of plasma or brain tissue, and secondly no cross-interference between G–A and internal standard using the proposed extraction procedure and HPLC conditions. Six different plasma samples were used as blank (G–A free plasma) were evaluated both with and without internal standard to assess the specificity of method.

### 3.13. Accuracy and Precision

The intra- and inter-assay accuracies were expressed as the percentage difference between the measured concentration and the nominal concentration. The intra-assay precision and accuracy were calculated using replicate determinations (*n* = 6) for each concentration of G–A added to plasma sample during a single analytical run. The inter-assay precision and accuracy were calculated using replicate determinations (*n* = 6) for each concentrations of G–A made on three separate days. Accuracy was calculated using the following equation: (% Bias) = [(C_obs_–C_nom_) / C_nom_] × 100. The precision was calculated from the observed concentrations as follows: RSD = [standard deviation (SD) / C_obs_] × 100. Accuracy (Bias) and precision (RSD) values within ±15%, covering a range of actual experimental concentrations, were considered acceptable (7).

### 3.14. Recovery (Extraction Efficiency)

The extraction efficiency of G–A was determined by analyzing replicate sets (*n* = 6) of QC samples: 0.8, 4.0 and 10 μg/mL for mice plasma and brain, representing low, medium and high QCs, respectively. Recovery was calculated by comparing the peak areas of G–A added into blank samples and extracted using the protein precipitation procedure, with those obtained from G–A spiked directly into post-protein precipitation solvent at three QC concentration levels.

### 3.15. Stability Study

The stability of G–A in mice plasma and brain was assessed by analyzing replicates (*n* = 6) of QC samples at concentrations of 0.8, 4.0 and 10 μg/mL for mice plasma and brain. The investigation covered expected conditions during all of the sample storage and process periods, which included the stability data from freeze/thaw, bench-top, autosampler and long–term stability test. For all stability studies, freshly prepared and stability testing QC samples were evaluated by using freshly prepared standard curves for the measurement. The concentrations obtained from all stability studies were compared with the freshly prepared QC samples, and the percentage concentration deviation was calculated. The analytes were considered stable in mouse plasma and brain when the concentration difference between the freshly prepared samples and the stability samples was less than 15%.

### 3.16. Pharmacokinetic Analysis

To evaluate the suitability of the assay for pharmacokinetic studies, 200 mg/kg of G–A was orally administered to mice. Eight animals were used in each group different times (0, 5, 10, 15, 30, 60, 120, 240, 480, 720, and 1440 min). Pharmacokinetic calculations were performed using the observed data. All data were subsequently processed using PKSolver add-in program for Microsoft Excel written in Visual Basic for Applications [8]. All values obtained were expressed in mean ± standard deviation.

The pharmacokinetic model selection either one or two-comparment, were used both the Akaike information criterion (AIC) and Schwarz criterion (SC). Using the criteria, one may select the model with which to reach a lower value of AIC or SC, which means that the chosen model is more parsimonious (less parameters required) and best fits the data (low error prediction) [12]. 

## 4. Conclusions

The identification and quantification of the anxiolytic G–A in brain tissue in this work, supports the fact that this molecule is capable of accessing the CNS and act on its pharmacologic target(s).
